# New Frontiers in the Treatment of Multiple Myeloma

**DOI:** 10.1100/tsw.2006.236

**Published:** 2006-12-06

**Authors:** Janice Jin Hwang, Irene M. Ghobrial, Kenneth C. Anderson

**Affiliations:** ^1^ Harvard Medical School, Boston, MA, USA; ^2^ Jerome Lipper Multiple Myeloma Center, Department of Medical Oncology, Dana Farber Cancer Institute, Boston, MA, USA

**Keywords:** multiple myeloma, signaling pathways, novel therapy, interleukin-6 (IL-6), proteasome inhibitor, thalidomide

## Abstract

Recent leaps in elucidating the biology of myeloma, particularly the intracellular pathways and the complex interaction with the bone marrow microenvironment, have resulted in an unprecedented surge of novel, targeted therapies and therapeutic regimens. There are currently over 30 new agents being tested in the treatment of multiple myeloma (MM). Many of these are novel, targeted agents that have demonstrated significant efficacy and prolonged survival. In this review, we summarize the current understanding of the mechanisms of action of novel therapies being tested in the preclinical and clinical settings in MM. These include agents that act directly on the intracellular signaling pathways, cell maintenance processes, and cell surface receptors. Finally, we present the clinical responses to some of these agents when used alone or in combination in clinical trials of patients with MM. Indeed, MM has become a model disease for the development of novel, therapeutic agents.

### INTRODUCTION

The first case descriptions of multiple myeloma (MM), a plasma cell malignancy characterized by lytic bone lesions, anemia, hypercalcemia, and renal failure, occurred as early as 1844 in patients described as having “mollities ossium” (soft bones); at the time, leeches and therapeutic bleeding were common treatment options[1,2]. It was not until over a century later, in 1958, that melphalan was first reported as a successful treatment for myeloma[3]. Shortly afterwards, melphalan combined with prednisone (MP) achieved better results than melphalan alone, and MP remained the conventional regimen until the recent advances of therapy in MM[4]. Recent leaps in elucidating the biology of myeloma, particularly the intracellular pathways and the complex interaction with the bone marrow microenvironment, have resulted in an unprecedented surge of novel, targeted therapies and therapeutic regimens. There are currently over 30 new agents being tested in the treatment of MM. Many of these are novel, targeted agents that have demonstrated significant efficacy and prolonged survival. Indeed, there has been a paradigm shift in the treatment of MM in the last 5 years.

Here, we provide a brief summary of the pathophysiology of MM, emphasizing the important role of the bone marrow microenvironment, and describing the mechanisms and pathways associated with novel therapies. We will discuss the preclinical evidence supporting novel therapies that target intracellular signaling pathways (Table 1), cell maintenance processes (Table 2), and cell surface receptors (Table 3). Finally, we will discuss therapies currently showing great promise in clinical trials both as single agents, and more importantly, in combination.

**TABLE 1**
**Novel Agents Targeting Intracellular Signaling Pathways**

| Pathways | Novel Agent | Description | Status |
|---|---|---|---|
| PI3K/Akt | Perifosine | Alkylphosphocholine | Phase II |
| | Rapamycin | mTOR inhibitor | |
| | CCI-779 | | Phase II |
| | RAD-001 | | Phase II |
| | Enzastaurin | PKCβ inhibitor | Phase II |
| MEK/ERK | Tipifarnib | Farnesyltransferase inhibitor | Phase II |
| | L744832 | | |
| | Manumycin | | |
| | BMS-214662 | | |
| | Lonafarnib | | |
| p38 MAPK | SCIO-469 | p38 MAPK inhibitor | Preclinical |
| MAPK/JNK | Aplidin/plitidepsin | | Phase II |
| NF-κB | PS-1145 | IKK inhibitor | Preclinical |
| | Bortezomib | Proteasome inhibitor | Phase III/FDA approved |
| JAK/STAT | Atiprimod | Azaspirane | Preclinical |
| Apoptosis | Arsenic trioxide | | Phase II |
| | LPAAT inhibitor | | Preclinical |
| | 2-Methoxyestradiol | | Preclinical |
| | ABT-737 | Bcl-2 inhibitor | Preclinical |
| | CDDO-Im | Triterpenoid | Preclinical |
| | VX-944 | Inosine monophosphate dehydrogenase inhibitor | Preclinical |
| | PK1195 | Smac mimetic | Preclinical |
| | FTY720 | Sphingosine monophosphate inhibitor | Preclinical |
| | Etodolac | | Preclinical |
| Multiple pathways | Thalidomide | | Phase III |
| | Lenalidomide/Revlimid | | Phase III |
| | CC-4047/Actimid | | Phase III |

### PATHOGENESIS OF MULTIPLE MYELOMA

Although MM is usually defined as a malignancy of the plasma cell, there is strong evidence to suggest that the initial mutation may have occurred in a less-differentiated cell[5]. Malignant plasma cells are usually immature plasmablasts that exhibit numerous chromosomal abnormalities. While a full discussion of the initial pathogenesis of disease is beyond the scope of this review, it is important to note that early

**TABLE 2**
**Novel Agents Targeting Cell Maintenance Processes**

| Cell Maintenance Process | Novel Agent | Description | Status |
|---|---|---|---|
| Protein degradation | Bortezomib/Velcade | Proteasome inhibitor | FDA-approved phase III |
| | NPI-0052 | | Preclinical |
| | Tubacin | Aggresome inhibitor | Preclinical |
| Transcription | SAHA | HDAC inhibitor | Phase I |
| | NVP-LAQ824 | | Preclinical |
| | NVP-LBH589 | | Preclinical |
| Protein chaperoning | 17AAG | Hsp90 inhibitor | Phase I/II |
| Mitosis | GRN163 | Telomerase inhibitor | Preclinical |

**TABLE 3**
**Novel Agents Targeting Cell Surface Receptors**

| Cell Surface Receptors | Novel Agent | Description | Status |
|---|---|---|---|
| IL-6 | Anti-IL-6 mAbs | | Phase I |
| | Sant-7 | IL-6 superantagonist | Preclinical |
| bFGF | SU5402 | Small molecule tyrosine kinase inhibitors | Preclinical |
| | PD173074 | | |
| | PKC412 | | |
| | PRO-001 | Anti-FGFR3 antibody | Preclinical |
| IGF-1 | NVP-ADW742 | IGFR tyrosine kinase inhibitor | Preclinical |
| VEGF | Avastin/bevacizumab | Anti-VEGF antibody | FDA approved for metastatic colon cancer |
| | PTK787 | VEGFR tyrosine kinase inhibitor | Phase I |
| | SU5416 | | Phase II |
| | GW654652 | | Preclinical |
| TNF family | TRAIL/Apo2L | | Preclinical |
| | SGN-40 | CD40 ligand | Preclinical |
| TGF-β | SD-208 | TGF-βR tyrosine kinase inhibitor | Preclinical |
| CD20 | Rituximab | Anti-CD20 antibody | Preclinical |

chromosomal translocations result in the overexpression of several important oncogenes, including MMSET and FGFR3 (at 4p16), CCN D3 (at 6p21), CCB D1 (at 11q13), c-MAF (at 16q13), and MAFB (at 20q11)[6,7,8,9,10]. In particular, as high as 40% of MM cells have at least one of these mutations[11]. Eventually, malignant clones carrying these mutations progress and undergo further genetic insults leading to advanced disease. In particular, mutations in c-myc, N-Ras, and K-ras oncogenes have been implicated in later stages of myeloma pathogenesis, and these will be discussed later in this review.

Environmental factors play an equally important role in the progression of disease. The malignant plasma cells home to the bone marrow where subsequent interactions serve to facilitate the development and progression of disease. In particular, advances in understanding myeloma biology have implicated the phosphatidylinositol-3 kinase (PI3K)/Akt (also known as protein kinase B [PKB]), mitogen-activated protein kinase (MAPK), Janus kinase 2 (JAK2)/signal transducers and activators of transcription (STAT) 3, I-κB kinase (IKK)/nuclear factor κB (NF-κB), and Hsp90 signaling pathways as key culprits in the pathogenesis of disease (Fig. 1).

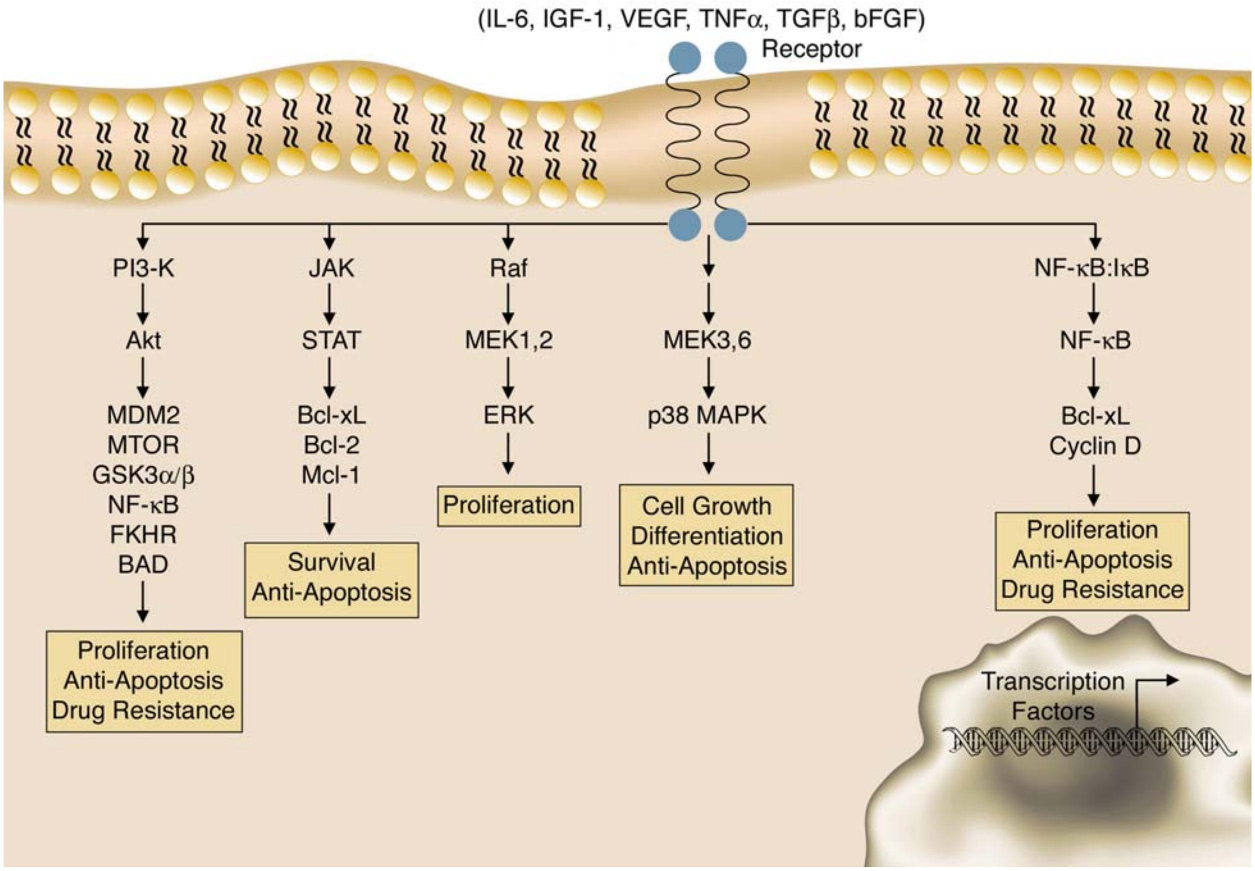

**FIGURE 1**. Simplified overview of intracellular signaling pathways.

The important role of the bone marrow microenvironment cannot be emphasized enough in the development of disease. There is now a large body of data describing how the bone marrow microenvironment, a complex landscape composed of hematopoietic stem cells, extracellular matrix (ECM) proteins, bone marrow stromal (BMSC) and endothelial cells, fibroblasts, osteoclasts, and osteoblasts, helps to facilitate MM cell growth, survival, and migration as well as mediate the development of drug resistance[12,13,14].

### NOVEL AGENTS TARGETING INTRACELLULAR SIGNALING PATHWAYS

Many of the agents currently being evaluated in myeloma exert their effects on a broad range of pathways, and for many drugs, the exact mechanisms of action are still being delineated. For the purposes of this review, the novel agents will be loosely grouped based on the pathways that appear to be most significant.

### Targeting the PI3K/Akt Pathway

The phosphatidylinositol-3 kinase (PI3K) signaling cascade is one of two major pathways that are activated by receptor tyrosine kinases. PI3K is composed of regulatory and catalytic subunits, which when activated, catalyze and further activate a wide range of downstream targets, most notably the serine/threonine protein kinase Akt (PKB). Akt has emerged as an important player in mediating tumor progression. It has a multifaceted role in cell survival, including sequestering the FOXO family of Forkhead transcription factors from activating their proapoptotic targets, such as FasL and Bim[15]; phosphorylating and thus sequestering Bad, a proapoptotic Bcl-2 family member, from the mitochondria[16]; phosphorylating I-κB kinase (IKK) and thus preventing the degradation of NF-κB[17]; and reciprocal regulation of the tumor suppressor gene, p53[18]. In addition, Akt regulates cell proliferation and growth by targeting the activity of glycogen synthase kinase β and preventing cyclin D1 degradation[19] as well as targeting mTOR (mammalian target of rapamycin). mTOR, also known as rapamycin-associated protein (FRAP), is a serine/threonine kinase that serves as a molecular sensor that regulates cell growth and proliferation in response to nutrients, growth factors, and insulin[20,21]. mTOR-dependent phosphorylation of several downstream molecules is critical for the cap-dependent translation of cell cycle proteins and progression from G1 to S phase[22].

Recently, there has been exciting evidence suggesting that the PI3K/Akt pathway is an important target in antimyeloma therapy. Akt is constitutively activated in patient myeloma cells, but interestingly, not in nonmalignant cells from the same patient[23]. Perhaps most importantly, many of the key growth factors in myeloma, such as IL-6, VEGF, and IGF-1, are ligands for tyrosine kinase receptors, which then activate the PI3K/Akt pathway. IL-6, the major myeloma growth factor, has been shown to induce phosphorylation of Akt and its downstream targets in a time- and dose-dependent manner. Furthermore, IL-6 overcomes dexamethasone-induced apoptosis via activation of PI3K/Akt[24].

Given the importance of the PI3K/Akt pathway in tumorigenesis, numerous drugs are currently under evaluation for a variety of malignancies[25]. In MM, three agents are currently being evaluated.

#### *Perifosine*

Perifosine, an orally active, alkyl-phosphocholine compound, belongs to a novel class of antitumor drugs that effect membrane permeability, phospholipid metabolism, and mitogenic signal transduction[26]. Importantly, perifosine has been shown to inhibit Akt activation without affecting the activity of PI3K or phosphoinositide-dependent kinase 1 (PDK1)[27]. It has been shown *in vitro* to induce $p21^{WAF1}$ expression and cell cycle arrest in head and neck squamous cell carcinoma[28], and two phase I studies have been completed in solid tumors[29].

In MM, Hideshima and colleagues found that perifosine inhibits Akt activation, induces JNK/caspase-dependent apoptosis in conventional therapy-resistant and -sensitive MM cell lines, overcomes the survival advantages of interaction between MM cell and BMSCs, and is not cytotoxic to peripheral blood mononuclear cells. Furthermore, it has significant antitumor effects in a murine MM mouse model[30]. Finally, given its pleotropic effects and based on initial preclinical studies, perifosine may have a valuable role in combination with other novel and standard therapies. When used in combination with standard therapies, such as dexamethasone, melphalan, and doxorubicin, perifosine augmented MM cytotoxicity *in vitro*. Interestingly, the proteasome inhibitor, bortezomib, was found to activate Akt and combination with perifosine blocked this activation. This finding offers preliminary evidence that combination with perifosine may overcome clinical resistance to bortezomib[30]. Phase II clinical trials are currently underway to evaluate the clinical uses for perifosine +/- dexamethasone as well as examining perifosine in combination with bortezomib in patients with relapsed or refractory MM.

#### *Rapamycin and Its Analogues: CCI-779, RAD-001*

Rapamycin and its more soluble derivatives CCI-779 and RAD-001 are mTOR inhibitors. Rapamycin binds to its receptor, FK506 binding protein12, which then complexes with mTOR to block its activity effectively[31,32]. Several studies have examined mTOR inhibitors' ability to not only induce cell cycle arrest, but induce apoptosis[32]. In particular, studies have found that both rapamycin and CCI-779 augment dexamethasone-induced apoptosis *in vitro* and *in vivo*[22]. mTOR inhibitors have been examined in phase II clinical trials for solid tumors, and are currently in phase II trials for myeloma[33]. In addition, Phase II trials of combination of CCI-779 with bortezomib are underway.

#### *Enzastaurin*

The protein kinase C (PKC) family of serine/threonine kinases has a myriad of targets that are involved in a broad range of cellular events, such as proliferation, growth, and transcription. Recent preclinical studies suggest that the PKCβ inhibitor, enzastaurin (LY317615), can overcome growth advantages conferred by BMSCs and acts on MM cell lines that are both sensitive and resistant to conventional therapies by inhibiting cell growth, survival, and migration[34]. Furthermore, enzastaurin may exert its apoptotic effects via inhibition of Akt[34,35]. A phase II trial of enzastaurin will be available for patients with relapsed/refractory MM.

### The MAPK Signaling Pathways

There are three major groups of mitogen-activated protein kinases (MAPKs): the extracellular signal-regulated kinase (ERK) family, the p38 MAPK family, and the c-Jun $NH_2$-terminal kinase (JNK) family. As their name implies, these families of serine/threonine kinases are activated by growth factors and other stimuli, and they also participate in the production and secretion of cytokines. The MAPKs are intimately involved in the regulation of key cellular processes, such as cell cycle progression, growth, differentiation, and apoptosis; as such, they are often implicated in malignant transformation and tumor progression[36].

#### *Targeting the Ras/Raf/MEK/ERK Family*

Numerous studies have shown that cytokine-induced cell proliferation is predominantly mediated through the ERK family of MAPKs[24,37,38,39,40], which are a part of a large cascade of proto-oncogenes including the upstream activators, Ras and Raf. In myeloma, 30–40% of patients have mutated Ras. N-Ras and K-Ras mutations, which lead to constitutively active Ras, have been observed in the malignant cells of patients with advanced stage disease[41,42]. While IL-6–dependent proliferation of MM cells is known to be dependent on ERK activation[43,44], interestingly, the inhibition of ERK in an IL-6–independent MM cell line with constitutively active Ras did not block cell proliferation. These data suggest that Ras can activate ERK-independent pathways[45].

##### Farnesyltransferase Inhibitor: Tipifarnib/R115777, L744832, Manumycin, BMS-214662, and Lonafarnib/SCH66336

There has been great interest in the use of farnesyltransferase inhibitors (FTIs) to target Ras in myeloma. Farnesyltransferase catalyzes the first step in post-translational modification of Ras, which allows the Ras protein to migrate to the cell membrane, where it exerts its activity. Several agents, including L744832[46], manumycin[47], BMS-214662[48], lonafarnib/SCH66336[49], and tipifarnib/R115777[50,51,52], have been shown to inhibit cell growth and survival in both drug-resistant and -senstitive MM cell lines. Of note, lonafarnib has been shown to work synergistically with bortezomib to enhance MM cell death. Interestingly, this synergistic response was associated with caspase cleavage as well as decreased phosphorylation of Akt, suggesting that FTIs may be promising agents in combination with proteasome inhibitors[49]. A phase II clinical trial of tipifarnib/R115777 in patients with advanced MM found that it was well tolerated and induced stabilization of disease[50].

### *Targeting the p38 MAPK Pathway*

p38 MAPK is a serine/threonine kinase involved in stress responses to environmental stressors such as inflammatory cytokines, UV light, and osmotic shock. There are four known splice variants of the p38 MAPKs, and p38α has been found to have major documented effects on cell growth, differentiation, and apoptosis[53].

#### SCIO-469

SCIO-469, a selective p38 MAPK inhibitor, has been studied in phase I/II trials for rheumatoid arthritis. In myeloma, it decreases proliferation of MM cells and blocks IL-6 and VEGF secretion from BMSCs[37]. In addition, SCIO-469 is being considered in combination with the proteasome inhibitor, bortezomib, because while bortezomib achieves excellent response in 35% of relapsed/refractory MM patients, there is a substantial population that are either unresponsive or develop resistance. SCIO-469 has been shown to augment the cytotoxicity of bortezomib *in vitro* and *in vivo*, in part due to down-regulation of Hsp27, a molecule whose overexpression has been associated with dexamethasone resistance[54,55].

### *Targeting the MAPK/JNK Pathway*

JNK, often found to be activated after chemotherapy, has been shown to exhibit both oncogene[56] and tumor-suppressor gene activities[57]. The exact role of JNK in myeloma is unclear; JNK inactivation appears to be one mechanism by which IL-6 protects MM cells from Fas-induced apoptosis[39,58], and the proteasome inhibitor, bortezomib, induces JNK activation during MM cell apoptosis[59,60]. The JNK1/2 specific inhibitor, SP600125, has been shown to induce G2/M arrest in MM cells and, interestingly, activates NF-κB in a time- and dose-dependent fashion[59].

#### Aplidin/Plitidepsin

Aplidin, a naturally occurring, cyclic depsipeptide isolated from the marine tunicate *Aplidium albicans*, exhibits very promising antitumor effects both *in vitro* and *in vivo*, and it is now in phase II clinical trials for a variety of solid and hematologic tumors. The exact mechanisms of action are unclear, but aplidin-mediated cytotoxicity has been shown to be dependent on sustained activation of JNK[61,62,63]. It exhibits strong apoptotic effects on MM cell lines and patient cells by triggering JNK, Fas, and mitochondrial-mediated signaling pathways[64].

Lopez-Martin and colleagues reported in abstract form that in Phase I and II trials of 215 and 112 patients, respectively, Aplidin was generally well tolerated with major dose limiting toxicity being adverse musculoskeletal events including increased CPK, myalgia and weakness [65]. Tumor shrinkage and long-lasting disease stabilization were reported in patients with colorectal, renal, neuroendocrine, lung, and head and neck cancer as well as melanoma and non-Hodgkin's lymphoma. Based on these initial results, phase II trials are currently underway in myeloma.

### Targeting the NF-κB Pathway

Nuclear factor κB (NF-κB), a small class of Rel family transcription factors, has emerged as a key player in the pathogenesis of myeloma. It plays a critical role in regulating many cellular responses, including immunity, inflammation, proliferation, survival, and angiogenesis[66,67,68]. Inactive NF-κB complexes with its inhibitor, IκBα, and remains sequestered in the cytosol. A variety of stimuli trigger the phosphorylation of IκB by IκB kinase (IKK). Phosphorylated IκB is then a target for ubiquination and proteasome-mediated degradation, which in turn releases NF-κB to translocate from the cytosol to the nucleus. Once in the nucleus, NF-κB stimulates transcription of numerous cytokines, chemokines, and cell adhesion molecules[69]. NF-κB is constitutively activated in numerous hematologic malignancies, including myeloma[70], and several agents have been examined to target the NF-κB pathway directly and indirectly.

#### *PS-1145*

PS-1145, a selective IKK inhibitor, was shown to inhibit IL-6 production from BMSCs cocultured with MM cells; however, compared to the complete cell proliferation blockade observed with bortezomib treatment, PS-1145 only partially inhibits MM cell proliferation *in vitro*, suggesting that there other therapeutic targets of proteasome inhibition[71,72,73].

#### *Other Agents*

Dexamethasone has been shown to down-regulate NF-κB activity, and conversely, constitutive activation of NF-κB mediates dexamethasone resistance in MM cells[74]. Thalidomide has also been shown to down-regulate NF-κB activity[75]. Finally, one of the most exciting new classes of agents in myeloma therapy is the proteasome inhibitor, which by preventing the degradation of NF-κB inhibitor, IκB, effectively inactivates NF-κB and prevents its promalignancy effects[76]. The prototype proteasome inhibitor, bortezomib, and its newer analogue, will be extensively discussed later in this review. Importantly, NF-κB blockade accounts for only part of bortezomib's antimyeloma effects.

### Targeting the JAK/STAT Pathway

The Janus kinase (JAK) family of tyrosine kinases plays a crucial role in cytokine signaling by phosphorylating the intracellular domains of cytokine receptors and recruiting downstream factors, such as STATs (signal transducers and activators of transcription), which then migrate to the nucleus and up-regulate gene transcription. STAT3 is of particular relevance in myeloma and other malignancies because its binding elements have been found on the promoters of several antiapoptotic genes, including Mcl-1, Bcl-2, and Bcl-xL[77]. Importantly, IL-6 binding to its receptor and the subsequent JAK/STAT3 activation is associated with myeloma cell survival[43,78] likely secondary to up-regulation of Mcl-1[79] and drug resistance[80].

#### *Azaspirane: Atiprimod*

Azaspiranes are compounds that have previously been studied in preclinical and clinical trials as anti-inflammatory agents for rheumatic diseases. While the exact mechanisms of action remain unclear, atiprimod, an orally bioavailable azaspirane, has been found to down-regulate the expression of adhesion molecules and cytokines, such as IL-6, TNF-α, and IL-2[81]. In myeloma, it has shown very promising *in vitro* antimyeloma effects including inducing apoptosis via down-regulation of phosphorylated STAT3 and its antiapoptotic targets, Mcl-1 and Bcl-2; inhibiting cell proliferation; and decreasing NF-κB activation. Importantly, the effects of atiprimod could not be overcome by the survival advantages conferred by IL-6, VEGF, or adherence of MM cells to BMSC[82]. Furthermore, azaspirane has been shown in a SCID-hu mouse model to inhibit tumor growth, further suggesting that it may have a beneficial role in patient therapy[83]. Currently, phase I/II trials are underway examining atiprimod in refractory or relapsed myeloma.

### Triggering Apoptotic Pathways

Almost all the therapies being evaluated in the treatment of myeloma exhibit some *in vitro* apoptotic effects, and many of these also have *in vivo* effects as well. The details of the intrinsic, extrinsic, and mitochondrial apoptotic pathways are beyond the scope of this review. In this section, the preclinical data for several novel therapies that have been shown to induce apoptosis in myeloma will be summarized, most notably, arsenic trioxide, LPAAT inhibitors, 2-methoxyestradiol, and others.

#### *Arsenic Trioxide ($As_2O_3$)*

Arsenic trioxide, currently being used in the clinic for treatment of relapsed/refractory, acute, promyelocytic leukemia, has shown promise in the treatment of myeloma as well[84]. *In vitro* studies have suggested that it works via several different pathways. Most notably, it has been shown to down-regulate Bcl-2, induce caspase-9 cleavage, and induce apoptosis in both drug-senstitive and -resistant MM cell lines[85]. Furthermore, it has been shown to inhibit both the JAK/STAT3 and NF-κB signaling pathways as well as decreasing paracrine IL-6 secretion from BMSCs[12,86]. It has also been found to up-regulate the expression of TRAIL (tumor necrosis factor–related apoptosis-inducing ligands) receptors, suggesting that combination with TRAIL may be of benefit[87].

#### *LPAAT Inhibitor*

Lysophosphatidic acid acyltransferase (LPAAT) catalyzes the conversion of lysophosphatidic acid to phosphatidic acid, a phospholipid involved in lipid biosynthesis and signal transduction. LPAAT-β inhibitors, in particular CT-32176 (the most potent), have been shown in myeloma cell lines to induce apoptosis via caspase cleavage and JNK signaling. Importantly, the antimyeloma effects of LPAAT-β inhibitors overcome growth advantages conferred by MM cell-BMSC adhesion and can overcome resistance to conventional therapies, such as dexamethasone and melphalan[88], as well as novel therapies, such as bortezomib[89], *in vitro*.

### *2-Methoxyestradiol*

2-Methoxyestradiol (2-ME2), an endogenous derivative of estradiol, was initially found to have significant antileukemic actions *in vitro* and *in vivo*. It has been found to induce apoptosis via the mitochondrial release of Smac protein and cytochrome C, which results in the inactivation of IAP (inhibitors of apoptosis) proteins followed by activation of the caspase cascade[90]. Furthermore, it decreases VEGF and IL-6 secretion from BMSC, suggesting antiangiogenic potential. In addition, *in vitro* and *in vivo* data indicate that 2-ME2 is effective in MM cells resistant to melphalan and doxorubicin[91].

### *Bcl-2 Inhibitor: ABT-737*

The Bcl-2 family of proteins is a key player in the regulation of mitochondria-dependent apoptosis. Overexpression of Bcl-2 and Bcl-xL has been noted in numerous malignancies, including MM, and Bcl-2 has also been implicated in the development of drug resistance and disease progression[92,93]. The small molecule Bcl-2 inhibitor, ABT-737, binds specifically to Bcl-2, Bcl-xL, and Bcl-w, and inhibits their proapoptotic effects by augmenting the effects of death signals[94].

Early results for ABT-737 have been very promising in solid tumors[94]. In myeloma, ABT-737 has been shown to induce apoptosis in MM cell lines and patient cells. Furthermore, it is cytotoxic in MM cells that are resistant to conventional therapies, and it enhances the antimyeloma effects of bortezomib and melphalan[93,95].

### *Others*

Many novel, apoptotic agents are currently in the early stages of evaluation in myeloma. Some of these include triterpenoids (CDDO-Im)[96], inosine monophosphate dehydrogenase inhibitors (VX-944)[97], Smac mimetics (PK1195), sphingosine monophosphate inhibitors (FTY720)[98], and etodolac (SDX-101)[99].

### Targeting Multiple Pathways: Thalidomide and Its Derivatives, Imids

Thalidomide, a glutamic acid derivative, was first used in myeloma based on its known antiangiogenic effects. However, it is now known that thalidomide and its more potent derivatives exert their activities through a broad spectrum of effects not limited to angiogenesis. In fact, microvessel density in the bone marrow and plasma VEGF and bFGF levels are not significantly different between patients treated with or without thalidomide[100,101]. Its other mechanisms of action include, but are not limited to, directly inhibiting MM cell growth and survival, preventing MM cell-BMSC adhesion, inhibiting secretion of cytokines needed for survival and growth, and promoting antimyeloma immune responses. The full details of the many cellular effects of thalidomide are beyond the scope of this review; however, several activities are particularly important in combating myeloma. Thalidomide blocks the secretion of potent myeloma growth factors (IL-6 and VEGF), induces apoptosis via caspase-8 activation, blocks NF-κB activity, and inhibits IL-6–induced MAPK pathways[12,102,103,104,105,106].

Thalidomide derivatives, such as lenalidomide (Revlimid)[107,108] and CC-4047 (Actimid) [109,110], are two of the most promising, second-generation thalidomide derivatives. Both can be administered orally and although they exert many similar biologic effects as thalidomide, both agents are much more potent and have significantly fewer toxic side effects than their predecessor[111,112,113]. Clinical trials are currently underway for both drugs and will be discussed later in this review.

### NOVEL AGENTS TARGETING CELL MAINTENANCE PROCESSES

#### Targeting Protein Degradation via Proteasomes and Aggresomes

##### *The Proteasome*

The ubiquitin-26S proteasome pathway, which regulates the turnover of a vast number of intracellular proteins, has become an exciting target in a variety of malignancies, most notably MM. Normally, proteins that are tagged with multiple, ubiquitin molecules enter the 26S proteasome for subsequent degradation. The proper functioning of this system is crucial for cell cycle regulation, gene transcription, and signal transduction. One of the proteins degraded by the 26S proteasome, IκBα, is an inhibitory protein that is bound to NF-κB and prevents NF-κB translocation to the nucleus. As mentioned earlier, once in the nucleus, NF-κB promotes the transcription of numerous genes involved in cell survival, proliferation, and drug resistance. Inhibition of the proteasome effectively increases the presence of IκBα and prevents NF-κB release to the nucleus.

###### Bortezomib

The prototype 26S proteasome inhibitor, bortezomib (Velcade, PS-341), selectively binds to the catalytic domain of the proteasome and prevents its activity. Predictably, bortezomib exhibited exciting antimyeloma effects associated with inhibition of NF-κB activity[72,76,114,115,116]; however, it should be noted that subsequent *in vitro* studies have revealed numerous other antimyeloma effects independent of the NF-κB pathway. Thus, the complete molecular mechanisms of its activity remain undefined[117]. It is now known to regulate cell cycle proteins, and targets both the intrinsic and extrinsic apoptotic pathways via caspase-9 and caspase-8, respectively.

###### NPI-0052

Recently, another proteasome inhibitor, NPI-0052, with a different chemical structure, toxicity profile, and mechanism of action, has been studied. Like bortezomib, NPI-0052 also inhibits NF-κB, blocks proteasome activity, and induces apoptosis in MM cells, but not BMSCs; furthermore, it is active at lower concentrations than bortezomib and can be orally administered. Interestingly, NPI-0052–mediated apoptosis appears to be predominately through the caspase-8 cell death cascade. This difference between bortezomib and NPI-0052 may, in part, explain the finding that a combination of the two proteasome inhibitors had a synergistic effect on cytotoxicity[118].

##### *The Aggresome*

There are several pathways through which misfolded or aggregated proteins are processed in a cell, including refolding via molecular chaperones or degradation via proteasomes. Recently, there has been evidence suggesting a third pathway of protein disposal involving the sequestration of aggregated proteins into spherical aggresomes for further processing, most commonly, lysosomal degradation. Notably, aggresomes are not simply static depositories for misfolded proteins, they also recruit chaperones and proteasomes to help in the clearance of the misfolded proteins. In light of the success of proteasome inhibition, it is possible that other means of cell protein catabolism may also be good therapeutic targets.

#### Tubacin

Histone deacetylase 6 (HDAC6) plays an essential role in aggresome activity by binding polyubiquinated proteins to the dynein motors needed for recruitment to aggresomes[119]. Tubacin, a small molecule that triggers acetylation of α-tubulin, directly inhibits HDAC6 to block aggresome activity. In a study by Hideshima and colleagues, tubacin was found to inhibit interaction with HDAC6. Furthermore, when used in combination with bortezomib, it induces synergistic accumulation of polyubiquinated proteins, increases cytotoxicity to MM cells, as well as decreases paracrine (BMSC)-induced cell growth[120].

### Targeting Transcription

Histones are positively charged proteins that attract and organize negatively charged DNA into nucleosomes. As such, their regulation by the opposing actions of histone acyltransferases and histone deacetylases (HDAC) plays a key role in gene expression, cell differentiation, and survival.

#### *HDAC Inhibitors: SAHA, NVP-LAQ824, and NVP-LBH589*

In MM, several novel HDAC inhibitors, including suberoylanilide hydroxamic acid (SAHA), NVP-LAQ824, and NVP-LBH589, are currently being evaluated. SAHA, in particular, has been shown to have pleiotropic antimyeloma effects; most notably, it induces apoptosis in MM cell lines and patient cells that are both sensitive and resistant to conventional therapies, and it sensitizes MM cells to other chemotherapies *in vitro*[121,122]. A phase I study of SAHA in relapsed/refractory myeloma is currently underway.

### Targeting Protein Chaperoning

Heat shock proteins (HSP) are a class of molecular chaperones that, under normal conditions, facilitate proper protein folding and regulate the turnover of important cell growth and survival proteins. When under conditions of environmental stress, HSP expression increases in an adaptive means to maintain cell homeostasis and enhance cell survival. Elevated levels of HSPs have long been noted in many malignancies, and these chaperones seem to help protect malignant cells from stressful microenvironments as well as from otherwise lethal mutations within the tumor cells themselves. HSP90 plays a particularly important role in oncogenesis because many of its protein substrates (such as receptor tyrosine kinases, serine/threonine kinases, telomerase, Akt, HIF1α) are signal transducers that regulate cell growth, proliferation, and survival[123,124].

#### *HSP90 Inhibitor: 17AAG*

Several HSP90 inhibitors have been tested in preclinical studies; however, only one, 17AAG, a geldanamycin derivative that binds to the N-terminal ATP-binding pocket of HSP90, has been studied *in vivo* and in clinical trials[125]. In myeloma, 17AAG induces potent apoptosis in drug-sensitive and -resistant cells *in vitro*. It has also been reported to act synergistically with bortezomib[126] and HDAC inhibitors[127]. Phase I trials are currently underway for 17AAG as a single agent in myeloma and also in combination with trastuzumab (Herceptin). Other HSP90 inhibitors with a more tolerable profile than 17AAG, such as KOS-953[128,129,130] and IPI-504[131,132,133], are being tested in phase I and II clinical trials in MM.

### Targeting Mitosis

Telomeres are nucleoprotein complexes that protect against degradation and erosion of chromosomes during replication cycles and serve to protect chromosome ends, which may otherwise be mistaken for double strand breaks, from fusion by repair mechanisms[134]. During each round of replication, the telomeres are eroded, and critical shortening of telomeres results in irreversible mitotic inhibition and cell death. Cells, thus, rely on the activity of telomerase, a reverse transcriptase, to lengthen and stabilize the telomeres, and in malignant cells, telomerase activity helps to confer immortality. High telomerase expression has been noted in up to 95% of human cancers[135,136].

#### *Telomerase Inhibitor: GRN163*

Telomerase inhibitors, such as GRN163[137], an oligonucleotide against human telomerase RNA component, and Telomestatin (3533-SV4)[138], an intercalating agent specific for telomeric sequences, have been found to shorten telomere length in MM cells and induce apoptotic cell death and growth inhibition.

## NOVEL AGENTS TARGETING CELL SURFACE RECEPTORS

### Targeting IL-6

IL-6 is known to be a major growth and survival signal in MM cells whose effects are both autocrine and paracrine[139,140]. Serum IL-6 levels correlate with the proliferative fraction of MM cells, and high levels are associated with a poor prognosis[141]. IL-6 is secreted by both tumor cells and BMSCs, and secretion is augmented by direct binding between tumor cells and BMSCs as well as by additional cytokines, such as TNFα, VEGF, and TGF-β, within the BM microenvironment[142].

IL-6 activates several major signaling cascades, including the Ras/Raf/MEK/ERK, the JAK2/STAT3, and the PI3K/Akt cascades, which mediate cell proliferation, survival, and drug resistance, respectively[12]. The initial step in the activation of these pathways involves the binding of IL-6 to its low-affinity receptor (IL-6Rα/gp80) and the subsequent homodimerization of signal transducer, gp130[143]. Notably, gp130 has no IL-6 binding capacity by itself, but activation by the IL-6/IL-6R complex results in homodimerization and phosphorylation of tyrosine residues in the intracellular domain of gp130 by the JAK family of enzymes[144,145].

#### *Anti-IL-6 Monoclonal Antibody*

Treatments targeting IL-6 have focused on monoclonal antibodies (mAbs) to IL-6 and IL-6R, and, more recently, the IL-6 superantagonist, Sant7. Anti-IL-6 mAbs, initially studied as promising therapies for rheumatoid arthritis and lupus, have been shown to have antitumor effects in animal and preclinical human studies[146,147]. In myeloma, anti-IL-6 mAbs have cytostatic effects on tumor cells *in vitro* as well as transient, antimyeloma effects in both animal models and human preclinical trials[148,149]. In particular, Bataille and colleagues found in a clinical trial that treatment with anti-IL-6 mAbs had antimyeloma effects, such as reduction of myeloma cell production and inhibition of C-reactive protein synthesis, an acute phase reactant synthesized in the liver in response to IL-6; however, none of the patients achieved remission or improvement as assessed by standard clinical criteria[150].

#### *IL-6 Superantagonist: Sant7*

Recently, much interest has turned to IL-6 superantagonists, which have a high affinity for IL-6R, but no bioactivity[151]. The most potent of these superantagonists, Sant7, has been shown to inhibit cell proliferation and induce apoptosis in IL-6–dependent myeloma cell lines[152]. Importantly, it has shown promise in combination with dexamethasone, one of the most active drugs in the treatment of MM. Numerous studies have implicated IL-6 production in the development of MM cell resistance to dexamethasone[104,153,154]. Sant7 overcomes resistance to dexamethasone in MM cell lines as well as potentiates the cytotoxic effects of dexamethasone and zoledronic acid[155,156]. Importantly, when evaluated in a SCID-hu *in vivo* mouse model of myeloma, Sant7 also significantly potentiates the antimyeloma effects of dexamethasone without significantly affecting CD34+ hematopoeitic progenitor cell growth[151]. Taken together, Sant7 is a promising therapeutic agent when used in combination with glucocorticoids, such as dexamethasone.

### Targeting Fibroblast Growth Factor

FGF-2 (basic FGF, bFGF) is a potent angiogenic cytokine secreted by MM cells and, to a lesser extent, BMSCs. Increased levels of FGF-2 are seen in serum, bone marrow, and plasma cell lysates of MM patients. Studies have demonstrated that serum FGF-2 levels decreased significantly after successful MM treatment[157,158,159]. Furthermore, paracrine interactions between FGF-2 and IL-6 contribute to increased neovascularization as well as MM cell proliferation. Notably, IL-6 enhances FGF-2 expression and secretion by MM cell lines and patient cells, and stimulation of BMSCs with FGF-2 induces a time- and dose-dependent increase in IL-6 secretion[157].

The signaling of FGF-2 is mediated by binding to a family of four distinct tyrosine kinase receptors (FGFR1-FGFR4), all of which are present on patient BMSCs and MM cells. Activation of FGFRs transduces signals through MAPK and PI3K pathways. Dysregulation of fibroblast growth factor receptor 3 (FGFR3) by the t(4;14) translocation is known to confer a poorer prognosis and is a primary event in 15–20% of MM cases[160,161]. Thus, there has been interest in targeting FGFR3 by both selective small molecule tyrosine kinase inhibitors and monoclonal antibodies.

#### *Small Molecule Tyrosine Kinase Inhibitors: SU5402, PD173074, PKC412*

Small molecule tyrosine kinase inhibitors, such as SU5402, PD173074, and PKC412, decrease viability and induce tumor cell growth arrest in human MM t(4,14) cell lines[162,163,164]. However, these agents may be limited by cross-reactivity with other receptor kinases.

#### *Anti-FGFR3 Antibody: PRO-001*

PRO-001, an anti-FGFR3 antibody, has a high affinity for FGFR3, decreases proliferation, and induces apoptosis in t(4:14) MM cells[165]. These preclinical studies suggest a role for FGFR3 inhibitors in MM patients with the t(4,14) translocation.

### Targeting Insulin-Like Growth Factor-1

IGF-1, a critical cytokine in the pathogenesis of MM, is known to have a plethora of downstream effects, including the activation of the MAPK/ERK and PI3K/Akt signaling pathways[166]. In recent studies, it has been shown to act synergistically with IL-6 and protects against dexamethasone-induced apoptosis[13,167,168]. Furthermore, IGF-1 mediates MM cell growth and survival in MM cells both *in*

*vitro*[168,169] and *in vivo*[170]. IGF-1 is a ligand for IGF receptor (IGF-1R), a tyrosine kinase signaling molecule, which is universally expressed on hematologic and solid tumor cell lines as well as patient MM cells[170].

#### IGFR Tyrosine Kinase Inhibitor: NVP-ADW742

Given the pleiotropic effects of IGF-1, targeted strategies against IGF-1R may have important clinical relevance [167]. A study by Mitsiades and colleagues showed that IGF-1R inhibition by the small-molecule IGF-1R tyrosine kinase inhibitor, NVP-ADW742, induced cytotoxicity in MM cells and was active even in cell lines resistant to conventional treatments, such as dexamethasone. Importantly, systemic administration of NVP-ADW742 suppressed tumor growth, prolonged survival, and potentiated the effects of other chemotherapies *in vivo*[170].

### Targeting Vascular Endothelial Growth Factor

VEGF, a potent angiogenic factor, is produced both by MM cells and BMSCs[171,172]. In addition to neovascularization, it has pleiotropic effects in the pathogenesis of MM, including aiding MM cell migration via PI3K-dependent PKCα activation, increasing proliferation and resistance to apoptotic signals via the up-regulation of Mcl-1, and augmenting the secretion of IL-6 by the BMSC[172,173,174,175]. The VEGF ligand exerts its effect after binding to its high-affinity tyrosine kinase receptor molecule, Flt-1, which is expressed on both MM patient cells as well as cell lines[171,172]. VEGF-triggered phosphorylation of Flt-1 activates the MAPK signaling pathway and ultimately leads to increased proliferation[12].

#### Bevacizumab (Avastin)

Agents targeting VEGF have shown great promise in the treatment of other malignancies. Most notably, the humanized monoclonal antibody against VEGF, bevacizumab (Avastin), was recently FDA approved as first-line therapy for metastatic colon cancer when given in combination with 5-FU[176]. Given the important role that VEGF plays in the progression of myeloma, these agents are now being studied as potential antimyeloma therapies. Bevacizumab is being studied in relapsed or refractory MM (with or without thalidomide).

#### VEGF Receptor Tyrosine Kinase Inhibitor: PTK787, SU5416, and GW654652

Therapeutic agents targeting VEGF receptor tyrosine kinase include PTK787, SU5416, and GW654652[177,178,179,180]. PTK787, an orally administered tyrosine kinase inhibitor that binds to the ATP-binding sites of VEGF receptors, has been shown *in vitro* to inhibit MM cell growth and migration as well as inhibit paracrine interactions with IL-6[177]. It is currently undergoing phase I testing in MM. SU5416, a small molecule VEGFR2, was found in phase II clinical trials to have some biologic effects; however, there was minimal clinical response[181]. Finally, the pan-VEGF inhibitor GW654652 acts on both MM cells and the BM microenvironment. GW654652 inhibits the secretion of other prominent cytokines (IL-6) and decreases proliferation even in the presence of BMSC. Phase I clinical trials are planned for the future[180].

#### Targeting the Tumor Necrosis Factor Family

The tumor necrosis factor family includes numerous ligands, several of which have been studied in MM, including TNF-α, tumor necrosis factor–related apoptosis-inducing ligand (TRAIL/Apo2L), Fas, CD40 ligand, B-cell activating factor, BAFF, and APRIL.

##### *TRAIL/Apo2L*

TRAIL/Apo2L appears to have the most potential benefit against myeloma as it has been shown in MM cell lines, MM patient cells, and in xenograft mouse models to induce apoptosis selectively and overcome drug resistance[182,183]. TRAIL binds to two receptors, TRAIL-R1 and TRAIL-R2, which then trimerize and ultimately trigger the activation of caspase cascade and apoptosis.

##### *CD40 Ligand: SGN-40*

CD40 ligand has been shown to effect MM cell proliferation directly via the PI3K/Akt pathway as well as indirectly through induction of IL-6 and VEGF secretion by the BMSCs[184]. Preclinical studies with a humanized anti-CD40 antibody, SGN-40, revealed cytotoxicity even in cell lines resistant to conventional therapies[185]. Furthermore, lenalidomide was shown to augment SGN-40–mediated cytotoxicity[107].

#### Targeting TGF-β

Transforming growth factor (TGF)-β1, a multifunctional cytokine that plays a major role in hematopoiesis and tumor progression, is known to enhance IL-6 secretion by BMSCs[142]. It is secreted predominantly by MM cells, and adhesion of MM cells with MM patient BMSCs augments this secretion[186].

##### *TGF-β Receptor Tyrosine Kinase Inhibitor: SD-208*

Inhibition of TGF-β1 may overcome the growth advantages conferred by MM cell adhesion to BMSCs. SD-208, a selective TGF-β receptor type I (TβRI) kinase inhibitor, down-regulates both cytokine secretion and proliferation of tumor cells even in the presence of BMSCs[186].

#### Anti-CD20 Antibody: Rituximab

CD20 is expressed on the cell surface in roughly 20% of MM patients, and a CD20+ phenotype is associated with shorter survival[187]. Rituximab, an anti-CD20 monoclonal antibody, is standard therapy for other hematologic malignancies, such as non-Hodgkin’s lymphoma. In myeloma, its clinical use is uncertain. It has been studied as a single agent[188,189], with modest results and in combination with melphalan/prednisone, also with equivocal results[190].

### NOVEL AGENTS CURRENTLY IN CLINICAL TRIALS

Many of the novel agents discussed in this review have shown great preclinical promise, both as single agents and in combination with current therapies. There are several upcoming or currently in progress clinical trials for many of these agents (Tables 1-3). Here we will briefly summarize the clinical data for several agents that have been more extensively studied: arsenic trioxide, thalidomide, lenalidomide, and bortezomib.

### Arsenic Trioxide

Arsenic trioxide has been evaluated in clinical trials as both a single agent and in combination with other therapies. A phase 2, multicenter, open-label study was conducted in 24 MM patients relapsed or refractory to prior treatments. Patients received arsenic trioxide (0.25 mg/kg/day for 5 day/week) during the first 2 weeks of each 4-week cycle; 58% had either a >25% reduction in serum M-protein levels or had stable disease[191]. Arsenic has also been evaluated in a combination study with dexamethasone and melphalan. In a study of 10 patients with relapsed or refractory disease, arsenic with low-dose melphalan and ascorbic acid exhibited sustained response and treatment was well tolerated[192]. Ascorbic acid potentiates the effects of arsenic by reducing intracellular glutathione, a molecule that functions to repair mitochondrial damage[193]. Currently, phase II trials are underway to evaluate arsenic in combination with bortezomib, thalidomide, and melphalan.

### Thalidomide

Thalidomide has shown excellent results as a single agent in patients with relapsed/refractory myeloma[101,194,195,196] and newly diagnosed disease[197,198,199]. Clinical response (complete, partial, and minor) was achieved in up to 50% of patients refractory to other treatments, and 30% of patients with new disease responded with 50% decreases in paraprotein. Subsequently, it has also been studied in combination with dexamethasone[195,199,200,201,202,203,204,205,206], dexamethasone/ cyclophosphamide[207,208,209,210], melphalan/prednisone[211], and as maintenance therapy following autologous stem cell transplantation[212,213,214].

Thalidomide treatment is associated with several treatment-limiting side effects, including neuropathy (50–80% of patients), venous thromboembolism (1–3% patients with Thal alone; 10–15% with thal/dex), Stevens-Johnson syndrome, and hepatotoxicity. Other side effects include fatigue, somnolence, constipation, and rash[113]. For this reason, newer, more potent, thalidomide immunomodulatory derivatives were developed with fewer side effects.

### Lenalidomide

Lenalidomide has been found *in vitro* to be as much as 2000 times more potent than thalidomide. Importantly, clinically it is much better tolerated with only rare neuropathy and reversible myelosuppression. Lenalidomide has also been studied in phase II trials as a single agent and in combination with dexamethasone for relapsed/refractory disease[215,216] as well as newly diagnosed disease[217]. Two large phase III trials of lenalidomide in combination with dexamethasone vs. dexamethasone alone in patients with relapsed/refractory myeloma have been recently presented at the American Society of Hematology annual meeting (2005) by Dimopoulos and colleagues[218]. Two large phase III trials of lenalidomide in combination with dexamethasone versus dexamethasone alone in patients with relapsed/refractory myeloma have been recently presented at the American Society of Hematology annual meeting (2005) by Dimopoulos and colleagues [219] and Clinical Trial number NCT00098475 [220].These have demonstrated significant activity of the lenalidomide dexamethasone arm with 58% response rate as compared to 22% in the dexamethasone arm. Based on these data, lenalidomide was recently approved in 2006 for use in patients who have received prior therapy. In addition, current clinical trials of a combination of lenalidomide with bortezomib in the upfront or relapsed setting are underway.

### Bortezomib

Bortezomib has seen a remarkable transition from bench to bedside. The SUMMIT trial (Study of Uncontrolled Multiple Myeloma managed with proteasome Inhibition Therapy), a large, multicenter phase II study in 2003, revealed a 35% response rate[220]. The CREST (Clinical Response and Efficacy Study of bortezomib in the Treatment of myeloma) trial, another phase II study randomizing patients to higher (1.3 mg/m3) or lower (1.0 mg/m3) doses of bortezomib in combination with dexamethasone, revealed positive response rates (33% with low-dose bortezomib alone, 44% with low-dose bortezomib/dex, 50% with high-dose bortezomib, and 62% with high-dose bortezomib/dex)[221]. Based on the results from these trials, bortezomib was FDA approved for treatment of relapsed and refractory myeloma in 2003. Subsequently, during interim analysis of an international, randomized, phase III trial of bortezomib vs. high-dose dexamethasone (APEX), bortezomib was found to be clearly superior in terms of overall survival and time to progression, and FDA approval was extended to include relapsed myeloma[222].

Currently, numerous phase I/II trials are underway to examine the effects of bortezomib as first-line therapy and in combination with other agents. Preliminary data from interim analysis in these trials are highly promising for an even greater role of bortezomib in the treatment of myeloma. In relapsed/refractory myeloma, bortezomib is being evaluated in combination with pegylated liposomal doxorubicin[223], melphalan[224], doxorubicin/thalidomide/dexamethasone[225], pegylated liposomal dox/low-dose dex[226], thalidomide and dex[227,228]. As a first-line agent, it is being evaluated in the phase III VISTA trial (Velcade as Initial Standard Therapy in multiple myeloma: Assessment with melphalan and prednisone), with dexamethasone[229], doxorubicin/dexamethasone (PAD)[230], and thalidomide and dexamethasone[231]. Other trials include the bortezomib/lenalidomide trial in newly diagnosed patients with MM.

### FUTURE DIRECTIONS: NOVEL DRUG COMBINATIONS

Treatments for MM have come a long way since therapeutic bleeding and leeches. Though myeloma remains an incurable disease, the recent decade has marked a renaissance in how myeloma is being studied and how new therapies are being developed. There has been a shift towards developing an arsenal of rationally designed, specific agents, each designed at targeting a small aspect of the complex disease. However, despite the advances observed in the treatment of myeloma, many patients still succumb to their disease. In addition, many agents that were exciting and promising in preclinical trials, fail to demonstrate similarly promising clinical activity as single agents in clinical trials. As such, one of the major challenges on the road towards improved survival and, perhaps, a cure, lies in the identification of not just promising agents, but combinations of agents. There is an urgent need for future clinical trials designed to combine novel agents rationally in order to achieve a higher response rate and longer remissions.

To date, there are already a vast number of *in vitro* and *in vivo* studies that hint at the myriad of pathways that can be targeted for a synergistic, multihit approach. Identifying these areas of molecular synergism depends on close collaboration between basic researchers at the bench and clinicians at the bedside, and will surely help to overcome drug resistance, extend patient survival, and improve quality of life.

### ACKNOWLEDGMENTS

KCA is supported in part by NIH grants RO-1 50947 and PO1-78378, Doris Duke Distinguished Clinical Research Scientist Award, and the Cure for Myeloma Fund. JJH is a Howard Hughes Medical Institute Medical Research Training Fellow. IMG is a Lymphoma Research Scholar and supported in part by the Leukemia and Lymphoma Society, an ASH Scholar award, and the Multiple Myeloma Research Foundation.
